# In Vitro Activity of Isavuconazole and Amphotericin B in Association against Mucorales

**DOI:** 10.3390/pathogens12070948

**Published:** 2023-07-18

**Authors:** Gaia Ortalli, Ester Oliva, Giuliana Lo Cascio, on behalf of the Medical Mycology Committee (CoSM)—Italian Association of Clinical Microbiologists (AMCLI), Claudio Farina

**Affiliations:** 1 Microbiology and Virology Laboratory, ASST “Papa Giovanni XXIII”, Piazza OMS 1, 24127 Bergamo, Italy; gaia.ortalli@apss.tn.it (G.O.); ester.oliva@ausl.re.it (E.O.); cfarina@asst-pg23.it (C.F.); 2 Microbiology and Virology Laboratory, Ospedale Guglielmo da Saliceto, Via Taverna 49, 29121 Piacenza, Italy; 3 Medical Mycology Committee (CoSM)—Italian Association of Clinical Microbiologists (AMCLI), Via Carlo Farini 81, 20159 Milano, Italy; segreteriaamcli@amcli.it

**Keywords:** Mucormycetes, Isavuconazole, Amphotericin B, association, *Epsilon test* synergy-method

## Abstract

Mucormycoses can be treated with the combination of Amphotericin B and Isavuconazole. This study evaluates the effects of these drugs in vitro against 59 strains representing 12 Mucorales. In vitro testing of the two drugs together and alone was performed using the MIC *Test strip* “*Epsilon test* synergy-method” (ETSM), which is more standard in clinical practice than microbroth dilution testing. Amphotericin B and Isavuconazole have synergistic/additive effects against *L. corymbifera*, *R. arrhizus* and *M. circinelloides*. Different effects have been shown for other Mucorales. ETSM can help the clinical management of mucormycosis from a practical point of view, due to its feasibility in the laboratory.

## 1. Introduction

Invasive mold infections are important causes of morbidity and mortality in immunocompromised hosts [1,2]. Mucoromycosis is a life-threatening fungal infection caused by fungi belonging to the subphylum *Mucoromycotina,* order Mucorales. The species commonly causing mucoromycosis belong to the genera *Rhizopus*, *Mucor*, *Lichtheimia*, *Cunninghamella*, *Syncephalastrum*, *Rhizomucor*, and others [3].

The European Confederation of Medical Mycology guideline for the management of mucormycosis suggests an early complete surgical treatment for mucoromycosis whenever possible, in addition to systemic antifungal treatment [4], even if a multimodal approach including association with the iron-chelator deferasirox administration [5], hyperbaric oxygen treatment [6], and granulocyte-macrophage colony-stimulating factor and/or interferon-γ adjunctive therapy [7] is only tentatively proposed [8,9].

Liposomal Amphotericin B (AMB) is the first line therapy to treat mucormycosis, even if AMB deoxycholate can be used only to treat patients when there is no other antifungal therapy available [10]. Conversely, Mucorales are known to be resistant to voriconazole in vitro and in vivo [11].

Isavuconazole (ISA) is an extended-spectrum antifungal triazole, offering advantages in terms of predictable pharmacokinetic/pharmacodynamic (PK/PD) safety profiles over the other mold-active triazoles, and in vitro activity against a wide variety of fungi, including *Candida* spp., non-*Aspergillus* spp., *Fusarium* spp., *Scedosporium* spp., and Mucorales. Specifically, isavuconazonium sulfate (the ISA prodrug formulation) may be orally or intravenously administered to patients with decreased renal function without the need for dose adjustment, due to the lack of cyclodextrin and minimal renal excretion [12,13]. The combined antifungal treatment using polyenes plus echinocandins or triazoles have not been recommended yet for first line therapy [14,15]. However, combination antifungal therapy can provide a potential strategy to improve antimicrobial activity and clinical outcomes, both in animal models and in humans [15,16,17]. 

Even if in vitro studies have evaluated the activity of azoles and polienes against yeasts [18,19] and *Aspergillus* [20,21,22], no studies are available concerning combinations of antifungal agents against Mucorales. 

The aim of this study was to test the in vitro effects of the AMB and ISA combination against clinical strains of the most common zygomycetes. 

## 2. Materials and Methods

We evaluated the MICs of 59 strains of Mucorales, isolated from clinical specimens: *Actinomucor elegans* (1), *Cunninghamella bertholletiae* (2), *Lichtheimia corymbifera* (12), *L. ramosa* (1), *Mucor circinelloides* (11), *M. hiemalis* (4), *M. indicus* (1), *M. racemosus* (4), *Rhizomucor* spp. (1), *R. microsporus* (1), *R. pusillus* (6), *Rhizopus arrhizus* (12), *Syncephalastrum racemosum* (3). All strains were identified using microscopy and confirmed by molecular method (sequencing internal transcribed spacer-ITS). *Paecilomyces variotii* (ATCC MYA 3630), *Aspergillus flavus* (ATCC 204304), and *A. fumigatus* (ATCC 204305) were used as quality control. 

In vitro testing of the two drugs, together and alone, was performed in duplicate using MIC Test strip “*Epsilon test* synergy-method” (ETSM), as described by the manufacturer (Liofilchem srl, Roseto degli Abruzzi, Italy). The combined effects of AMB and ISA were quantified after 24 h of incubation at 35 °C.

The Fractional Inhibitory Concentration (FIC) *Index* was determined as the MIC value of one molecule when the other was present as well [23]. It was calculated based on the following formula: FIC *Index* = MIC_AB_/MIC_A_ + MIC_BA_/MIC_B_. MIC_AB_ is the MIC of drug A in the presence of drug B (ISA in the presence of AMB); MIC_A_ is the single MIC of drug A; MIC_BA_ is the MIC of drug B in the presence of drug A (AMB in the presence of ISA); MIC_B_ is the single MIC of drug B.

The interpretation, in therapeutic categories, considers that an FIC *index* ≤ 0.5 is synergic; >0.5–≤1 is additive; >1–≤4 is indifferent; >4 is antagonist. 

## 3. Results

For all the Mucorales species tested in this study, the MIC ranged between 0.19 μg/mL and 32 μg/mL for ISA and between 0.047 and 32 μg/mL for AMB tested alone.

Table 1 shows the in vitro susceptibility of the 59 Mucorales, tested individually against AMB and ISA, as detailed above.

As shown in Table 2, the effect of the association of AMB and ISA significantly varied according to the different genera and species of the Mucorales tested. 

None of the *C. bertholletiae* isolates showed any interaction between the two antifungal agents; similarly, two out of three *S. racemosum* isolates were defined as indifferent, while the third showed an additive effect between ISA and AMB. Among *L. corymbifera*, only three strains showed synergistic effect, while nine had an additive effect. Concerning *R. pusillus*, four isolates were indifferent to the association with ISA and AMB, one strain showed an additive effect and synergic effect only one. 

Three different types of effects were detected testing *R. arrhizus*: the association between ISA and AMB had additive effect on eight of the twelve strains; there was a synergistic effect on three strains and no effect on one isolate upon drug combination. 

Among the eleven isolates of *M. circinelloides*, the combination of the two antifungals showed an additive effect on seven isolates, no effect on three, and only one strain showed synergistic effect.

Regarding *M. racemosus*, an indifferent effect was detected on two strains, and on the other two isolates, an additive effect was observed. Among *M. hiemalis*, the combination showed a synergistic effect for three strains, and indifferent for one isolate. 

*Lichtheimia ramosa* showed a synergistic effect; for *R. microsporus*, the interaction between the two drugs was always additive.

## 4. Conclusions

This study provides preliminary evidence that the combination of ISA and AMB shows high variability, according to the genus and the species of the Mucorales tested. For this reason, identification is a mandatory priority also when the in vitro effects of two drugs in association must be investigated. These data confirm Borman et al.’s conclusions, who highlight the importance of fungal identification to propose the optimal treatment of Mucoromycoses [17]. As shown by our data, this is particularly relevant for those genera where the effects of the AMB and ISA in association varies drastically from isolate to isolate, as in *R. arrhizus* and *R. pusillus,* where the first species showed a prevalent synergistic effect, and the latter showed mainly an indifferent effect. Furthermore, it is worth noting that the in vitro association test against *L. corymbifera* and *R. arrhizus*, the most frequent isolated species of Mucorales, substantially confirms an additive or synergistic effect, while for *C. bertholletiae*, the effect is always indifferent for unknown reasons.

In conclusion, this study confirms that not only the in vitro MIC distributions of AMB and ISA alone against Mucorales are species-dependent, but also the effect of their associations, even if PK/PD interactions should be studied with conventional in vitro tests [24]. It provides additional data on the challenging therapy options against mucoromycetes, as combination antifungal therapy can provide a potential strategy to improve clinical outcomes. This study presents the first in vitro evidence of the effects of the association of two antifungals against Mucorales, evaluated by the Gradient Concentration Strip Method. Even if deviates from the standard microdilution checkerboard methodology, P Vidal et al. reported that the essential agreement within ±2 dilution steps at 24 hours between these techniques was 83.3% and 73.3% for ISA and AMB, respectively [25,26]. However, ETSM can help the clinical management of mucoromycosis from a practical point of view, due to its feasibility in the laboratory, adapting the technical procedure proposed by A Espinel-Ingroff et al. for Mucorales [27].

## Figures and Tables

**Table 1:** In vitro susceptibilities of *Mucorales* tested individually with ISA and AMB.

	ISA	AMB
*Actinomucor elegans (1)*	3	0.75
*Cunninghamella bertholletiae (2)*	1	0.032–0.19
*Lichtheimia corymbifera (12)*	0.19–6	0.094–2
*Lichtheimia ramosa (1)*	32	0.125
*Mucor circinelloides (11)*	2–32	0.047–32
*Mucor hiemalis (4)*	0.75–32	0.004–0.032
*Mucor indicus (1)*	8	0.064
*Mucor racemosus (4)*	0.75–32	0.125–1
*Rhizomucor* sp. *(1)*	0.75	0.19
*Rhizomucor microsporus (1)*	0.75	0
*Rhizomucor pusillus (6)*	0.38–6	0.064–32
*Rhizopus arrhizus (12)*	0.094–2	0.047–32
*Syncephalastrum racemosum (3)*	0.75–32	0.004–0.47

**Table 2:** Effects of the combination of ISA and AMB with the relative Fractional Inhibitory Concentration *Index* (FIC).

Samples	MIC ISA with AMB	MIC AMB with ISA	FIC Index	Effect
*Actinomucor elegans*	1.5	0.25	0.833	ADDITIVE
*Cunninghamella bertholletiae*	1	0.125	1.658	INDIFFERENT
	1	0.064	3	INDIFFERENT
	0.75	0.008	0.875	ADDITIVE
	3	0.19	0.595	ADDITIVE
	0.5	0.023	0.851	ADDITIVE
	1.5	0.047	0.747	ADDITIVE
	0.19	0.012	0.628	ADDITIVE
*Lichteimia corymbifera*	0.25	0.047	0.594	ADDITIVE
	0.125	0.008	0.828	ADDITIVE
	0.25	0.047	0.313	SYNERGIC
	0.19	0.094	1	ADDITIVE
	0.094	0.023	0.217	SYNERGIC
	0.5	0.094	0.625	ADDITIVE
	0.19	0.023	0.374	SYNERGIC
*Lichtheimia ramosa*	4	0.012	0.221	SYNERGIC
	3	0.75	1	ADDITIVE
	8	0.016	0.590	ADDITIVE
	32	0.38	1.380	ADDITIVE
	32	0.047	2	INDIFFERENT
	2	32	2	INDIFFERENT
*Mucor circinelloides*	3	0.023	0.583	ADDITIVE
	6	0.047	0.688	ADDITIVE
	1.5	0.032	0.750	ADDITIVE
	0.75	0.016	0.358	SYNERGIC
	0.5	0.047	1.250	INDIFFERENT
	3	0.023	0.583	ADDITIVE
	0.75	0.016	0.38	SYNERGIC
*Mucor hiemalis*	4 0.125	0.032 0.064	1.13 0.50	INDIFFERENT SYNERGIC
	1.5	0.004	0.50	SYNERGIC
*Mucor indicus*	3	0.032	0.875	ADDITIVE
	32	0.19	1.380	ADDITIVE
*Mucor racemosus*	32 32	0.19 1	1.380 2.000	ADDITIVE INDIFFERENT
	0.38	0.094	1.259	INDIFFERENT
*Rhizomucor* sp.	0.25	0.047	0.747	ADDITIVE
*Rhizomucor microsporus*	0.19	0.002	0.753	ADDITIVE
	0.125	0.016	0.457	SYNERGIC
	6	0.064	2	INDIFFERENT
	0.5	1	1	ADDITIVE
*Rhizomucor pusillus*	1.5	0.094	2	INDIFFERENT
	1.5	0.19	2.771	INDIFFERENT
	32	0.75	2	INDIFFERENT
	0.19	0.006	0.888	ADDITIVE
	0.38	1.5	1	ADDITIVE
	0.38	0.012	0.519	ADDITIVE
	0.38	0.25	0.802	ADDITIVE
	2	0.5	1	ADDITIVE
*Rhizopus arrhizus*	0.75 1	0.5 32	1 3.632	ADDITIVE INDIFFERENT
	0.064	0.003	0.175	SYNERGIC
	0.19	0.006	0.198	SYNERGIC
	0.25	8	0.750	ADDITIVE
	0.064	0.006	0.729	ADDITIVE
	0.125	0.032	0.465	SYNERGIC
	0.7	0.06	2.36	INDIFFERENT
*Syncephalastrum racemosum*	1	0.04	1.5	INDIFFERENT
	0.19	0.002	0.753	INDIFFERENT

## Data Availability

The datasets generated during and/or analyzed during the current study are available from the corresponding author on reasonable request.
